# New Evidence of Potential Benefits of Dexamethasone and Added on Therapy of Fludrocortisone on Clinical Outcomes of Corticosteroid in Sepsis Patients: A Systematic Review and Meta-Analysis

**DOI:** 10.3390/jpm11060544

**Published:** 2021-06-11

**Authors:** Ji-young Son, Sooyoung Shin, Yeo Jin Choi

**Affiliations:** 1 Department of Clinical Pharmacy, Graduate School of Pharmacy, CHA University, Seongnam 13488, Korea; a01077218649@gmail.com; 2 Department of Clinical Pharmacy, College of Pharmacy, Ajou University, Suwon 16499, Korea; 3 Research Institute of Pharmaceutical Science and Technology (RIPST), Ajou University, Suwon 16499, Korea; 4 Department of Clinical Pharmacy, Graduate School of Clinical Pharmacy, CHA University, Seongnam 13488, Korea

**Keywords:** corticosteroid, dexamethasone, fludrocortisone, mortality, sepsis, septic shock

## Abstract

The aim of this study is to investigate clinical outcomes of corticosteroid treatment in patients with sepsis or septic shock. An electronic keyword searches of PubMed, EMBASE, and Google Scholar were conducted per PRISMA guidelines. The pooled analyses on the corticosteroid impact on mortality, adverse events, and clinical outcomes were performed. Subgroup analyses on the clinical outcomes in relation to corticosteroid dose, duration, and agents were performed. Pooled analyses of 21 randomized control trials revealed substantially reduced mortality (RR 0.93, 95% CI 0.88–0.99, *p* = 0.02) and length of stay in intensive care unit (SMD −1.66, 95% CI −1.91–−1.40, *p* < 0.00001) without increased risks of adverse events (RR 1.04, 95% CI 0.96–1.12, *p* = 0.38). No significant improvements of other clinical outcomes were observed. Subgroup analyses demonstrated substantially reduced mortality with short-term (≤7 days) low-dose (<400 mg/day) corticosteroid treatment (RR 0.91, 95% CI 0.87–0.95, *p* < 0.0001). Moreover, dexamethasone (RR 0.40, 95% CI 0.20–0.81, *p* = 0.01) and combined hydrocortisone and fludrocortisone treatment (RR 0.89, 95% CI 0.84–0.94, *p* < 0.00001) provided substantial reduction of mortality whereas hydrocortisone alone did not reduce the mortality risk in sepsis patients. Thus, further controlled studies on the clinical outcomes of potential corticosteroid options on sepsis-related clinical outcomes are warranted.

## 1. Introduction

Sepsis is classified as systemic inflammatory responses to infection manifested by innate immune system activation, which subsequently induces life-threatening organ dysfunction or septic shock [1]. The number of incident sepsis steadily increases each year, affecting approximately 48.9 million patients worldwide [1]. The deleterious features of sepsis involving responses from vascular, immune, platelets, and plasma protein substantially increase the risk for mortality, which is estimated to be 30–50% [2]. Sepsis is considered as the leading cause of in-hospital death, and 11.0 million sepsis-related mortality was reported in 2017 [2]. However, underestimation of sepsis-related mortality is anticipated as data on incidence and mortality of sepsis in low- and middle-income countries are limited [1].

The major pathophysiological components of sepsis include cytokine-mediated inflammation, endothelial injury, vasodilation, and hypercoagulability; nevertheless, the recommended treatment modalities mainly include broad-spectrum antibiotics for infection, fluid resuscitation, along with vasopressor to ameliorate hemodynamic imbalance [2,3]. The current guideline, Surviving Sepsis Campaign, published by Society of Critical Care Medicine and European Society of Intensive Care Medicine, encourages intravenous antibiotic initiation within an hour of recognition of sepsis or septic shock, as early antibiotic administration reduces infection-mediated inflammation, thereby improving survival [4]. A previous study also revealed 7.6% reduction in survival for every hour of delay in antibiotic initiation [4,5]. Nonetheless, the sepsis-related mortality still remains high despite appropriate treatment, implying the need for discovery of therapeutic agent that may improve clinical outcomes in sepsis patients. 

The current guideline recommends low dose (<400 mg, typically 200–300 mg/day) hydrocortisone only in sepsis patients with adrenal insufficiency or refractory hypotension defined as systolic blood pressure <90 mmHg notwithstanding appropriate fluid resuscitation and vasopressor treatment [4,6,7]. However, previous studies evaluating corticosteroid-related clinical outcomes in sepsis patients provided controversial results regardless of strong immunosuppressive anti-inflammatory activity [8,9,10]. Moreover, these studies recruited critically-ill patients with diagnosis other than sepsis, which may impede clinical applications of these results. Therefore, the objective of this study is to evaluate clinical outcomes of corticosteroids in sepsis and septic shock patients by performing pooled analyses of double-blinded, placebo-controlled randomized trials investigating efficacy and safety of corticosteroids.

## 2. Materials and Methods

### 2.1. Search Strategy and Study Selection

This study was prepared according to Preferred Reporting Items for Systematic Reviews and Meta-Analyses (PRISMA) guidelines [11]. A systematic literature search of PubMed, Embase, and Google Scholar was performed to identify randomized clinical trials evaluating clinical outcomes including efficacy and safety of corticosteroid in sepsis patients (from inception to October 2020). The methods of initial database search include a combination of keywords and Medical Subject Headings including ‘corticosteroids’, ‘steroids’, ‘sepsis’, and ‘septic shock’ in title/abstracts. Reference lists of studies eligible for full-text review were further screened to identify eligible studies. Two reviewers (JS and YJC) searched electronic databases and identified eligible clinical trials, and any disagreements regarding study selection were resolved by the third person (Shin). The eligibility of studies was determined by prespecified inclusion criteria: (1) patients aged >17 years who had primary diagnosis of sepsis or septic shock, (2) double-blinded, placebo-controlled randomized controlled trials (RCTs) comparing clinical outcomes of corticosteroid (intervention) over comparator (placebo), (3) studies that assessed outcomes of interests, and (4) studies published in English. Review articles, meta-analyses, duplicate studies, conference abstracts, proceedings, case reports, editorials, studies without full-texts, and studies written in languages other than English were excluded. Additionally, any studies that recruited patients with primary diagnoses other than sepsis such as acute respiratory distress syndrome (ARDS) or systemic inflammatory response syndromes (SIRS) were excluded. The primary outcomes of interest include mortality defined as death after randomization and adverse events (AEs) including gastrointestinal bleeding, hyperglycemia, and secondary infection after corticosteroid treatment. The secondary outcomes of interest include duration of mechanical ventilation, organ failures, respiratory failures, length of stay in hospital or intensive care unit (ICU), and reversal of shock. Two reviewers extracted study characteristics including first author; publication year; intervention regimen, duration, and dose; comparator; patient inclusion criteria; and outcomes of interest, and the doses of corticosteroids were converted into hydrocortisone equivalent dose. 

### 2.2. Risk of Bias Assessment 

The risk of bias assessment of included studies was evaluated by Cochrane Risk of Bias [12], and studies were scored as low, unclear, or high in the following features: randomization sequence generation, allocation concealment, blinding of participants and personnel, blinding of outcome assessment, incomplete outcome data, selective reporting, and other potential bias such as differences in baseline characteristics. Any disagreements on the study quality assessment were discussed until a consensus was reached. The funnel plots and Egger’s test were utilized to detect publication bias: a symmetric funnel plot and *p* > 0.05 from Egger’s test imply a low risk of publication bias. 

### 2.3. Statistical Analysis 

Pooled analyses of the outcomes of interest were conducted using RevMan (Review Manager Version 5.4, The Nordic Cochrane Center, The Cochrane Collaboration, Copenhagen, Denmark, 2020). The effect size of continuous variables such as length of stay in hospital or ICU and duration of mechanical ventilation were presented as weight standard mean differences (SMD) with 95% confidence intervals (CIs). The dichotomous variables including mortality, organ failure, respiratory failure, reversal of shock, and AEs were evaluated with relative risks (RR) and 95% CIs. Studies that measured mortality at multiple time points were analyzed as separate mortality cases. I^2^ index was utilized to determine heterogeneity across the studies, and Mantel–Haenszel fixed-effect model was used to analyze outcomes with low heterogeneity (I^2^ < 50%) while the random-effect model was performed to analyze outcomes with high heterogeneity (I^2^ > 50%) [13]. Subgroup analyses were performed to identify factors affecting corticosteroid-related clinical outcomes and 2 factors were analyzed: (1) treatment regimen in consideration of treatment duration (≤7 days or >7 days) and corticosteroid dose (hydrocortisone equivalent dose <400 mg/day or ≥400 mg/day) and (2) corticosteroid regimens including hydrocortisone, hydrocortisone with fludrocortisone, dexamethasone, methylprednisolone, and prednisolone. Any studies not meeting the criteria of subgroup analyses were excluded from the analyses. *p*-values were estimated by two-sided tests and any *p*-values < 0.05 were considered statistically significant. 

## 3. Results

### 3.1. Study Selection and Characteristics

**Figure 1:**
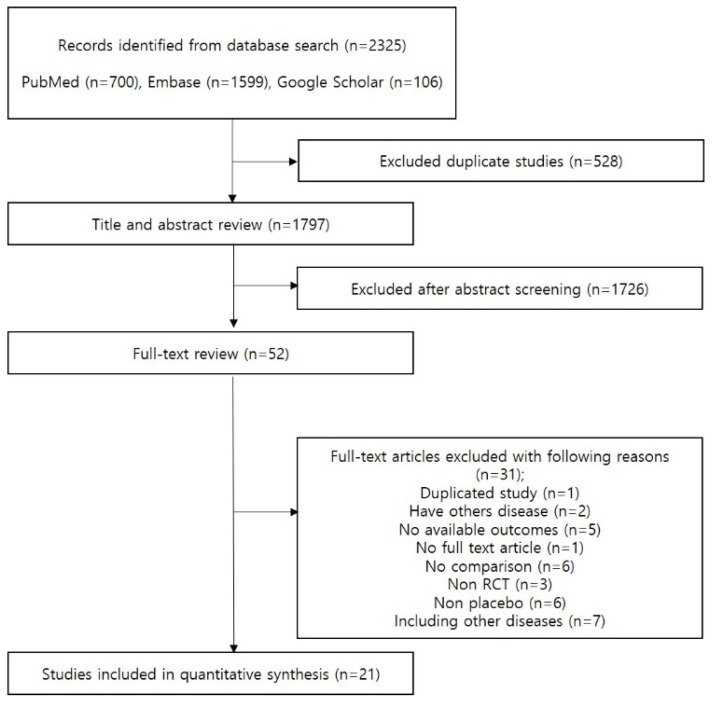
PRISMA Plot.

The primary database search yielded 2325 studies, and 52 studies were eligible for full-text review (Figure 1). Thirty-one studies were excluded after full text-review and a total of 21 RCTs evaluating the effects of corticosteroids in 8127 sepsis patients (4054 on corticosteroid and 4073 on placebo) were included in the analysis. The study characteristics of eligible studies are described in Table 1. Thirteen studies administered hydrocortisone [14,15,16,17,18,19,20,21,22,23,24,25,26], two studies administered hydrocortisone and fludrocortisone [27,28], two studies administered dexamethasone [29,30], and four studies administered methylprednisolone [30,31,32,33], and two studies administered prednisolone [34,35]. All patients included in this analysis were diagnosed with sepsis [17,19,20,21,24,25,28,31,33,34,35] or septic shock [14,15,16,18,20,22,23,24,26,27,28,29,30,32]. The quality assessment results are described in Supplementary Table S1 and the risk of bias was generally acceptable as implied by symmetric funnel plots and Egger’s test results (*p* > 0.05 for all outcomes) (Supplementary Figure S1).

### 3.2. Clinical Outcomes

**Figure 2:**
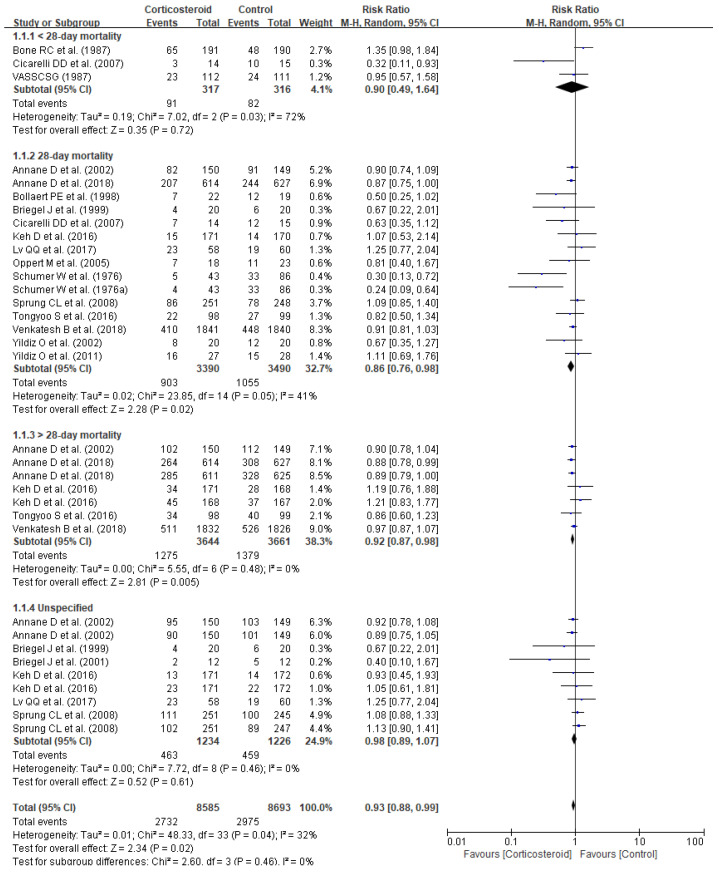
Forest plot of corticosteroid impact on mortality.

**Figure 3:**
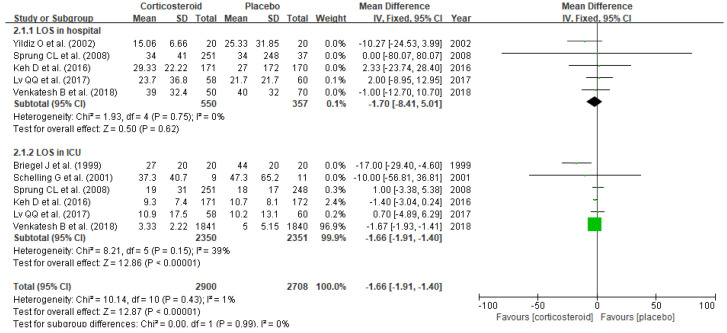
Forest plot of length of stay in hospital and ICU.

Corticosteroid treatment substantially reduced mortality (RR 0.93, 95% CI 0.88–0.99, *p* = 0.02), especially 28-day mortality (RR 0.86, 95% CI 0.76–0.98, *p* = 0.02) and long-term mortality defined as >28-day mortality (RR 0.92, 95% CI 0.87–0.98, *p* = 0.005) in patients diagnosed with sepsis or septic shock (Figure 2). Corticosteroid also reduced the length of stay in ICU (SMD −1.66, 95% CI −1.91–− 1.40, *p* < 0.00001) (Figure 3). Meanwhile, no substantial benefits of other clinical outcomes including length of stay in hospital (SMD −1.70, 95% CI −8.41–5.01, *p* = 0.62), organ failure (RR 1.02, 95% CI 0.66–1.59, *p* = 0.93), respiratory failure (RR 1.01, 95% CI 0.89–1.14, *p* = 0.88, reversal of shock (RR 0.91, 95% CI 0.79–1.05, *p* = 0.18), and mechanical ventilation duration (SMD −0.58, 95% CI −2.64–1.47, *p* = 0.58) were noticed (Table 2).

### 3.3. Subgroup Analyses

**Figure 4:**
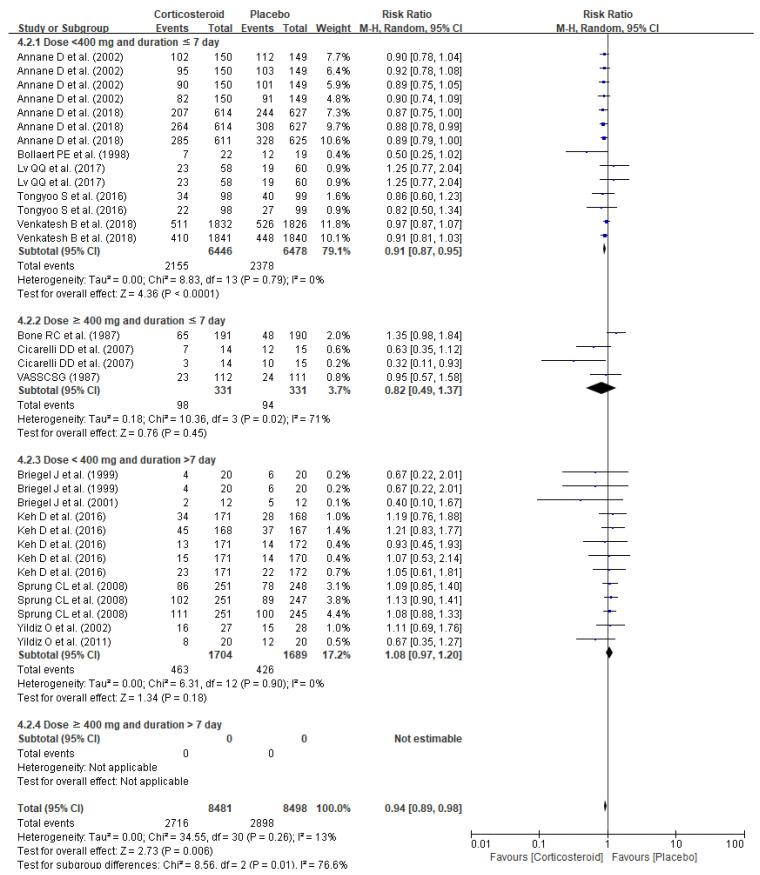
Forest plots of subgroup analysis of mortality per corticosteroid treatment duration and dose.

**Figure 5:**
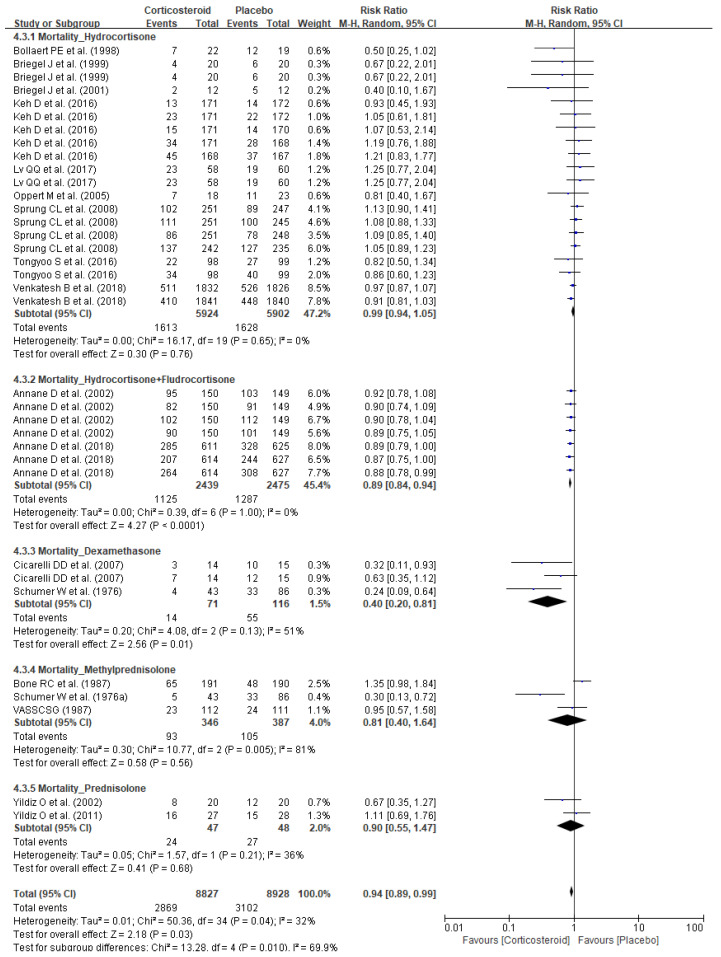
Forest plot of subgroup analysis on impact of corticosteroid agents on mortality.

The pooled analysis of dose and duration demonstrated markedly lowered mortality with short-term (≤7 days) low-dose (<400 mg/day) corticosteroid (RR 0.91, 95%CI 0.87–0.95, *p* < 0.0001) whereas no substantially improved survival was observed with other treatment plans: short-term (≤7 days) high-dose (≥400 mg/day) corticosteroid (RR 0.82, 95% CI 0.49–1.37, *p* = 0.45) and long-term (>7 days) low-dose (<400 mg/day) corticosteroid (RR 1.08, 95% CI 0.97–1.20, *p* = 0.18) (Figure 4). The impacts on mortality risk differed among corticosteroid agents, and only hydrocortisone and fludrocortisone (RR 0.89, 95% CI 0.84–0.94, *p* < 0.0001) and dexamethasone (RR 0.40, 95% CI 0.20–0.81, *p* = 0.01) provided substantial reduction in mortality risks, whereas no changes in mortality risks were observed with hydrocortisone alone (RR0.99, 95% CI 0.94–1.05, *p* = 0.76), methylprednisolone (RR 0.81, 95% CI 0.40–1.64, *p* = 0.56), and prednisolone (RR 0.90, 95% CI 0.55–1.47, *p* = 0.68) (Figure 5).

### 3.4. Adverse Events

**Figure 6:**
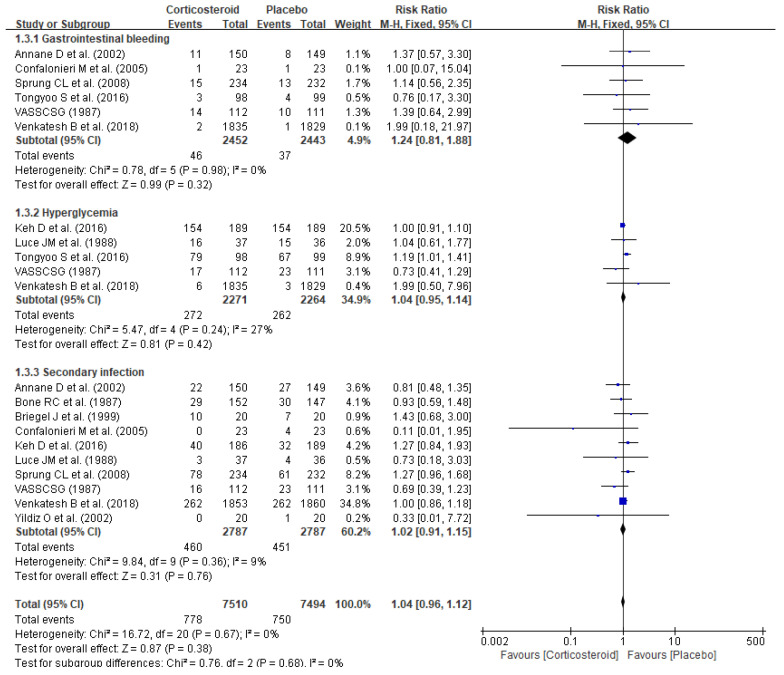
Forest plot of adverse events.

No significant elevation of corticosteroid-related AE risks (RR 1.04, 95% CI 0.96–1.12, *p* = 0.38) including gastrointestinal bleeding (RR 1.24, 95% CI 0.81–1.88, *p* = 0.32), hyperglycemia (RR 1.04, 95% CI 0.95–1.14, *p* = 0.42), and secondary infections (RR 1.02, 95% CI 0.91–1.15, *p* = 0.76) were observed (Figure 6).

## 4. Discussion

This study investigated corticosteroid-related clinical outcomes in patients diagnosed with sepsis or septic shock. Corticosteroid treatment substantially reduced mortality (RR 0.93, 95% CI 0.88–0.99, *p* = 0.02), especially 28-day mortality (RR 0.86, 95% CI 0.76–0.98, *p* = 0.02) and >28-day mortality (RR 0.92, 95% CI 0.87–0.98, *p* = 0.005) without elevated risks of AEs (RR 1.04, 95% CI 0.96–1.12, *p* = 0.38) in sepsis patients. The subgroup analysis revealed improved survival in patients diagnosed with sepsis or septic shock with short-term (≤7 days) low-dose (<400 mg/day) corticosteroid therapy (RR 0.91, 95% CI 0.87–0.95, *p* < 0.0001). Moreover, substantially lowered mortality rates were observed only with added on therapy of fludrocortisone to hydrocortisone (RR 0.89, 95% CI 0.84–0.94, *p* < 0.00001) and dexamethasone (RR 0.40, 95% CI 0.20–0.81, *p* = 0.01).

The current guideline recommends low-dose (<400 mg/day) hydrocortisone treatment for ≥ 3 days (typically 200 to 300 mg/day for 5 to 7 days in real practice) in sepsis or septic shock patients who have refractory hypotension despite appropriate fluid resuscitation and vasopressor administration, and routine corticosteroid therapy is not suggested [4,7]. Our study results demonstrated similar results of previous meta-analyses which demonstrated reduced 28-day mortality with low-dose corticosteroid (<400 mg/day) [8,9], and another study also displayed a linear relationship between mortality and corticosteroid dose administered in the first 24 h after study enrollment [36], implying high-dose corticosteroid administration may result in more harm than benefits. Moreover, our study suggested that longer corticosteroid treatment (>7 days) does not guarantee improved survival, as cytokine-mediated inflammations are most substantial during the early phase of sepsis [1]. However, caution is advised with the determination of the actual duration of short-term (≤7 days) corticosteroid therapy at this point despite the recommendation of the guidelines (≥3 days) because a previous meta-analysis revealed improved survival with corticosteroid treatment duration ≥ 4 days [9].

This study demonstrated interesting results in relation to corticosteroid agents. Similar to the results of previous studies [8,9], combination therapy of hydrocortisone and fludrocortisone substantially reduced mortality of sepsis or septic shock patients. However, the current guideline recommends hydrocortisone as the glucocorticoid of choice in sepsis patients because, in addition to glucocorticoid effects, hydrocortisone also provides sufficient mineralocorticoid activity [4]. Notably, our meta-analysis findings also revealed that hydrocortisone combined with fludrocortisone, a major mineralocorticoid agent, improved survival outcomes in sepsis patients. Furthermore, we observed markedly improved survival with dexamethasone treatment (RR 0.40, 95% CI 0.20–0.81, *p* = 0.01), whereas hydrocortisone did not reduce mortality (RR 0.99, 95% CI 0.94–1.05, *p* = 0.76) in sepsis patients. The advantageous aspects of dexamethasone in sepsis patients may include the greatest glucocorticoid potency and anti-inflammatory activity among corticosteroids and dexamethasone has even higher anti-inflammatory activity than non-steroidal anti-inflammatory drugs [37]. The interest in dexamethasone has skyrocketed recently because of a study which demonstrated that 10-day treatment of low-dose dexamethasone (6 mg daily, hydrocortisone equivalent dose of 160 mg/day) substantially reduced 28-day mortality in hospitalized COVID-19 patients who required oxygen treatment [38], implying low-dose dexamethasone may play a strong drug candidate for improving survival of critically-ill patients. However, a question on the safety of dexamethasone in sepsis or septic shock patients must be answered because all studies evaluating dexamethasone effects administered a high hydrocortisone-equivalent dose (≥400 mg) in sepsis patients, and comprehensive analysis of clinical outcomes including efficacy and safety associated with low-dose (<400 mg/day) dexamethasone administration in sepsis patients is warranted due to the limited number of studies.

As far as we know, this is the first meta-analysis investigating corticosteroid impact on sepsis-related clinical outcomes from double-blinded, placebo-controlled RCTs. Moreover, another distinct feature of this study from previous meta-analyses is the inclusion of study populations with primary diagnosis of sepsis or septic shock [8,9]. Previous meta-analyses demonstrated controversial results on corticosteroid impact on mortality [8,9]. A meta-analysis of clinical outcomes of corticosteroid in pediatric and adult sepsis patients displayed insignificant short-term (28–31 day) mortality (RR 0.93, 95% CI 0.84–1.03, *p* = 0.15) [8], whereas a meta-analysis of adult sepsis patients revealed significantly improved 28-day mortality (RR 0.90, 95% CI 0.83–0.98, *p* = 0.02) [9]. However, these meta-analyses included studies that recruited patients with primary diagnoses other than sepsis or septic shock such as ARDS, SIRS, and community-acquired pneumonia [8,9], and data from patients without sepsis or septic shock diagnosis were also analyzed, which may impede clinical application of the study results in sepsis patients. This study demonstrated substantially reduced sepsis-related mortality with corticosteroids, however, subgroup analyses provide variable results in relation to treatment duration, dose, and corticosteroid agents, providing evidence that a hydrocortisone and fludrocortisone combination regimen and dexamethasone can be promising agents for improving survival in sepsis patients. However, further controlled studies on the clinical outcomes including efficacy and safety of dexamethasone in sepsis or septic shock patients are warranted.

This study possesses some limitations. First, heterogeneity across the studies may hinder clinical applications of this study. The included studies had variable study designs and outcome measurements. Moreover, although the included studies recruited patients with primary diagnosis of sepsis or septic shock, the conventional treatment may vary among the patients because of diverse underlying causes of sepsis including etiologic microorganisms. Additionally, sepsis may induce multiple organ failures and patient managements are guided based on the clinical presentations. To minimize the potential issues with heterogeneity and publication bias, our study team only analyzed the double-blinded, placebo-controlled RCTs. Additionally, inclusion of studies only published in English may raise concerns pertaining to limitation on study selection and strength of outcomes. To prevent the loss of evidence, our study group performed additional screening for the studies written in languages other than English and identified no eligible studies for the analysis, implying the strong validity of our study design and outcomes. Another concerning aspect is related to unassessed patient-related risk factors for poor prognosis of sepsis and corticosteroid-related AEs in the original studies, which may cause treatment response variabilities in these patients. Moreover, this study did not show any improvements in other clinical outcomes such as length of stay in hospital, organ failures, respiration failures, duration of mechanical ventilations, and reversal of shock due to limited number of studies. Nonetheless, this study possesses significant implication as survival is a critical indicator of sepsis-related clinical outcomes considering mortality rate of 30–50% in these patients, and our study team demonstrated improved survival with corticosteroid treatment in sepsis patients and identified factors that may benefit corticosteroid therapy. However, further studies on patient-specific factors related to variations in corticosteroid responses in sepsis patients to promote clinical benefits of corticosteroid therapy are needed.

## 5. Conclusions

Corticosteroid significantly reduced mortality in sepsis or septic shock patients. The pooled analyses revealed markedly reduced mortality with short-term (≤7 days) low-dose (<400 mg/day) corticosteroid treatment. Combination therapy of hydrocortisone and fludrocortisone and dexamethasone can be a promising therapeutic option to improve survival outcomes in sepsis patients.

## Figures and Tables

**Table 1:** Characteristics of the 21 clinical trials included in the meta-analysis on clinical outcomes of corticosteroids.

Author (Year)	Treatment (N)	Control (N)	Inclusion Criteria	Dose	Hydrocortisone Equivalent (mg/Day)	Duration (Day)	Outcomes
Bollaert PE et al., (1998) [14]	Hydrocortisone (N = 22)	Placebo (N = 19)	Septic shock requiring catecholamine for >48 h	300 mg IV/day	300	<5	Mortality
Briegel J et al., (1999) [15]	Hydrocortisone (N = 20)	Placebo (N = 20)	Adult patients who met ACCP/SCCM criteria for septic shock	100 mg (loading dose within 30 min) followed by a continuous infusion of 0.18 mg/kg/h (sepsis) or 0.08 mg/kg/h (septic shock) for 6 days	359.2	4–8	Mortality Secondary infection Mechanical ventilation LOS in ICU
Confalonieri M et al., (2005) [17]	Hydrocortisone (N = 23)	Placebo (N = 23)	Adult patients diagnosed with sepsis	200 mg (IV bolus) followed by 10 mg/h for 7 days	440	7	Mortality LOS in ICU LOS in hospital Mechanical ventilation ARDS Secondary infection Respiratory failure Organ failure Gastrointestinal bleeding
Briegel J et al., (2001) [16]	Hydrocortisone (N = 12)	Placebo (N = 12)	Patients with septic shock	Infusion of 100 mg of hydrocortisone, followed by 0.18 mg/kg/h (continuous infusion)	359.2	<6	Mortality
Kaufmann I et al., (2008) [18]	Hydrocortisone (N = 15)	Placebo (N = 15)	Patients admitted to ICU and met criteria for septic shock	100 mg (IV bolus), followed by 10 mg/h (continuous infusion)	340	1	Organ failure Respiratory failure
Keh D et al.,/HYPRESS study (2016) [19]	Hydrocortisone (N = 171)	Placebo (N = 172)	Sepsis patients >18 years	200 mg/day (continuous infusion) for 5 days, 100 mg (day 6 and 7), 50 mg (day 8 and 9), on days 8 and 9, and 25 mg (day 10 and 11)	200	5–11	Mortality LOS in ICU LOS in hospital Mechanical ventilation Secondary infection Respiratory failure Organ failure
Lv QQ et al., (2017) [26]	Hydrocortisone (N = 58)	Placebo (N = 60)	Age 18 years old or older, onset of septic shock within 6 h	200 mg/day	200	6	Mortality LOS in ICU LOS in hospital Reversal of shock
Moreno R et al.,/CROTICUS (2011) [20]	Hydrocortisone (N = 251)	Placebo (N = 248)	Patients >18 years diagnosed with sepsis or septic shock	50 mg (IV bolus every 6 h for 5 days), 50 mg (IV every 12 h for days 6–8), 50 mg (IV every 24 h for days 9–11)	200	11	Organ failure Respiratory failure
Oppert M et al., (2005) [21]	Hydrocortisone (N = 18)	Placebo (N = 23)	Adult patients met criteria for sepsis	50 mg (IV bolus) followed by 0.18 mg/kg body of weight/h (continuous infusion)	309.2	No record	Mortality
Schelling G et al., (2001) [22]	Hydrocortisone (N = 9)	Placebo (N = 11)	Adult patients with hyperdynamic septic shock	100 mg IV, 0.18 mg/kg/h	359.2	6	LOS in ICU Respiratory failure
Sprung CL et al., (2008) [23]	Hydrocortisone (N = 251)	Placebo (N = 248)	Adults septic shock patients	50 mg of IV every 6 h for 5 days; dose-tapering for 6 days	200	11	Mortality LOS in ICU LOS in hospital Secondary infection Respiratory failure Reversal of shock Gastrointestinal bleeding Organ failure
Tongyoo S et al., (2016) [24]	Hydrocortisone (N = 98)	Placebo (N = 99)	Age ≥18 years meeting the criteria for severe sepsis or septic shock	50 mg per 6 h (200 mg/day)	200	7	Mortality Mechanical ventilation Mechanical ventilation-free time Secondary infection Hyperglycemia Gastrointestinal bleeding
Venkatesh B et al., (2018) [25]	Hydrocortisone (N = 1853)	Placebo (N = 1860)	Sepsis adult patients (>18 years)	200 mg/day	200	≤7	Mortality Resolution of shock Reversal of shock LOS in ICU LOS in hospital Mechanical ventilation-free time Secondary infection Gastrointestinal bleeding
Annane D et al., (2002) [28]	Hydrocortisone and Fludrocortisone (N = 150)	Placebo (N = 149)	Adults (18 years or older) and hospitalized in ICU with sepsis/septic shock	Hydrocortisone (50 mg IV bolus every 6 h) and fludrocortisone (50 µg tablet once daily)	200.5	7	Mortality Secondary infection Gastrointestinal bleeding
Annane D et al., (2018) [27]	Hydrocortisone and Fludrocortisone (N = 614)	Placebo (N = 627)	Indisputable or probable septic shock patients	Hydrocortisone 50 mg IV every 6 h, fludrocortisone 50 μg tablet/day for 7 days	200.5	7	Mortality Mechanical ventilation
Cicarelli DD et al., (2007) [29]	Dexamethasone (N = 15)	Placebo (N = 15)	Septic shock patients aged ≥18 years and admitted to ICU	0.2 mg/kg IV at intervals of 36 h (total 3 doses)	640	4.5	Mortality Mechanical ventilation
Schumer W et al., (1976) [30]	Dexamethasone (N = 43)	Placebo (N = 86)	Septic shock	3 mg/kg	480	No record	Mortality
	Methylprednisolone (N = 43)	Placebo (N = 86)	Septic shock	30 mg/kg	900	No record	Mortality
Bone RC et al., (1987) [31]	Methylprednisolone (N = 191)	Placebo (N = 190)	Adult patients with infection plus the presence of fever or hypothermia, organ dysfunction	30 mg/kg × 4 doses	36,000	1	Mortality Reversal of shock Secondary infection
Luce JM et al., (1988) [32]	Methylprednisolone (N = 38)	Placebo (N = 37)	Patients with septic shock and ARDS	30 mg/kg, 1800 mg/60 kg × 4 doses	36,000	1	ARDS Total mortality Hyperglycemia Secondary infection
VASSCSG (1987) [33]	Methylprednisolone (N = 112)	Placebo (N = 111)	Systemic sepsis patients	30 mg/kg followed by infusion of 5 mg/kg	22,500	1	Mortality
Yildiz O et al., (2002) [34]	Prednisolone (N = 20)	Placebo (N = 20)	>17 years old and sepsis	5 mg IV at 06:00 am and 2.5 mg IV at 18:00 for 10 days	30	10	LOS in hospital Secondary infection Mortality
Yildiz O et al., (2011) [35]	Prednisolone (N = 27)	Placebo (N = 28)	Patients >17 years and diagnosed with sepsis	20 mg/day	80	10	Mortality

Abbreviations: ARDS: acute respiratory distress syndrome, CORTICUS: The corticosteroid therapy of septic shock, HYPRESS: The Hydrocortisone for Prevention of Septic Shock, ICU: intensive care unit, IV: intravenous LOS: length of stay.

**Table 2:** The corticosteroid impact on other clinical outcomes in sepsis patients.

	Outcome	Statistical Method	Studies	Participants	I^2^ (%)	Effect Estimate	*p*
Organ failure	Organ failure	Risk Ratio (M-H, Fixed, 95% Cl)	1	466	N/A	1.02 (0.66, 1.59)	0.93
	Respiratory failure	Risk Ratio (M-H, Fixed, 96% Cl)	5	1381	0	1.01 (0.89, 1.14)	0.88
Mechanical ventilation		Mean Difference (IV, Fixed, 95% Cl)	2	69	0	−0.58 (−2.64, 1.47)	0.58
Reversal of shock		Risk Ratio (M-H, Random, 95% Cl)	2	362	0	0.91 (0.79, 1.05)	0.18

Abbreviation: ARDS: Acute respiratory distress syndrome, ICU: intensive care unit, LOS: length of stay, NA: not applicable.

## Supplementary Materials

The following are available online at https://www.mdpi.com/article/10.3390/jpm11060544/s1, Table S1: Quality assessment of included studies of randomized controlled trials, Figure S1: Funnel plots of the study outcomes; (a) mortality and (b) adverse events.

## References

[1] Hotchkiss R.S., Moldawer L.L., Opal S.M., Reinhart K., Turnbull I.R., Vincent J.L. (2016). Sepsis and septic shock. Nat. Rev. Dis. Primers.

[2] Ladha E., House-Kokan M., Gillespie M. (2019). The ABCCs of sepsis: A framework for understanding the pathophysiology of sepsis. Can. J. Crit. Care Nurs..

[3] Rello J., Valenzuela-Sánchez F., Ruiz-Rodriguez M., Moyano S. (2017). Sepsis: A Review of Advances in Management. Adv. Ther..

[4] Rhodes A.A., Evans L.E., Alhazzani W., Levy M.M., Antonelli M., Ferrer R., Kumar A., Sevransky J.E., Sprung C.L., Nunnally M.E. (2017). Surviving Sepsis Campaign: International Guidelines for Management of Sepsis and Septic Shock: With supraphysiologic doses of hydrocortisone. Crit. Care Med..

[5] Mok K., Christian M.D., Nelson S., Burry L. (2014). Time to Administration of Antibiotics among Inpatients with Severe Sepsis or Septic Shock. Can. J. Hosp. Pharm..

[6] Annane D. (2016). The Role of ACTH and Corticosteroids for Sepsis and Septic Shock: An Update. Front. Endocrinol..

[7] Annane D., Pastores S.M., Rochwerg B., Arlt W., Balk R.A., Beishuizen A., Briegel J., Carcillo J., Christ-Crain M., Cooper M.S. (2017). Guidelines for the diagnosis and management of critical illness-related corticosteroid insufficiency (CIRCI) in critically ill patients (Part I): Society of Critical Care Medicine (SCCM) and European Society of Intensive Care Medicine (ESICM) 2017. Intensive Care Med..

[8] Rochwerg B., Oczkowski S., Siemieniuk R.A., Menon K., Szczeklik W., English S., Agoritsas T., Belley-Cote E., D’Aragon F., Alhazzani W. (2017). Corticosteroids in sepsis: An updated systematic review and meta-analysis (protocol). BMJ Open.

[9] Fang F., Zhang Y., Tang J., Lunsford L.D., Li T., Tang R., He J., Xu P., Faramand A., Xu J. (2019). Association of corticosteroid treatment with outcomes in adult patients with sepsis: A systematic review and meta-analysis. JAMA Intern. Med..

[10] Coutinho A.E., Chapman K.E. (2011). The anti-inflammatory and immunosuppressive effects of glucocorticoids, recent developments and mechanistic insights. Mol. Cell. Endocrinol..

[11] Moher D., Liberati A., Tetzlaff J., Altman D.G. (2009). Preferred Reporting Items for Systematic Reviews and Meta-Analyses: The PRISMA Statement. Ann. Intern. Med..

[12] Higgins J.P., Altman D.G., Gøtzsche P.C., Jüni P., Moher D., Oxman A.D., Savovic J., Schulz K.F., Weeks L., Sterne J.A. (2011). The Cochrane Collaboration’s tool for assessing risk of bias in randomised trials. BMJ.

[13] Higgins J.P.T., Thompson S.G., Deeks J.J., Altman D.G. (2003). Measuring inconsistency in meta-analyses. BMJ.

[14] Bollaert P.-E., Charpentier C., Levy B., Debouverie M., Audibert G., Larcan A. (1998). Reversal of late septic shock with supraphysiologic doses of hydrocortisone. Crit. Care Med..

[15] Briegel J., Forst H., Haller M., Schelling G., Kilger E., Kuprat G., Hemmer B., Hummel T., Lenhart A., Heyduck M. (1999). Stress doses of hydrocortisone reverse hyperdynamic septic shock: A prospective, randomized, double-blind, single-center study. Crit. Care Med..

[16] Briegel J., Jochum M., Gippner-Steppert C., Thiel M. (2001). Immunomodulation in septic shock: Hydrocortisone differentially regulates cytokine responses. J. Am. Soc. Nephrol..

[17] Confalonieri M., Urbino R., Potena A., Piattella M., Parigi P., Puccio G., Della Porta R., Giorgio C., Blasi F., Umberger R. (2005). Hydrocortisone infusion for severe community-acquired pneumonia: A preliminary randomized study. Am. J. Respir. Crit. Care Med..

[18] Kaufmann I., Briegel J., Schliephake F., Hoelzl A., Chouker A., Hummel T., Schelling G., Thiel M. (2008). Stress doses of hydrocortisone in septic shock: Beneficial effects on opsonization-dependent neutrophil functions. Intensive Care Med..

[19] Keh D., Trips E., Marx G., Wirtz S.P., Abduljawwad E., Bercker S., Bogatsch H., Briegel J., Engel C., Gerlach H. (2016). Effect of hydrocortisone on development of shock among patients with severe sepsis: The HYPRESS randomized clinical trial. JAMA.

[20] Moreno R., Sprung C.L., Annane D., Chevret S., Briegel J., Keh D., Singer M., Weiss Y.G., Payen D., Cuthbertson B.H. (2011). Time course of organ failure in patients with septic shock treated with hydrocortisone: Results of the Corticus study. Intensive Care Med..

[21] Oppert M., Schindler R., Husung C., Offermann K., Gräf K.-J., Boenisch O., Barckow D., Frei U., Eckardt K.-U. (2005). Low-dose hydrocortisone improves shock reversal and reduces cytokine levels in early hyperdynamic septic shock. Crit. Care Med..

[22] Schelling G., Briegel J., Roozendaal B., Stoll C., Rothenhäusler H.-B., Kapfhammer H.-P. (2001). The effect of stress doses of hydrocortisone during septic shock on posttraumatic stress disorder in survivors. Biol. Psychiatry.

[23] Sprung C.L., Annane D., Keh D., Moreno R., Singer M., Freivogel K., Weiss Y.G., Benbenishty J., Kalenka A., Forst H. (2008). Hydrocortisone Therapy for Patients with Septic Shock. N. Engl. J. Med..

[24] Tongyoo S., Permpikul C., Mongkolpun W., Vattanavanit V., Udompanturak S., Kocak M., Meduri G.U. (2016). Hydrocortisone treatment in early sepsis-associated acute respiratory distress syndrome: Results of a randomized controlled trial. Crit. Care.

[25] Venkatesh B., Finfer S., Cohen J., Rajbhandari D., Arabi Y., Bellomo R., Billot L., Correa M., Glass P., Harward M. (2018). Adjunctive glucocorticoid therapy in patients with septic shock. N. Engl. J. Med..

[26] Lv Q.-Q., Gu X.-H., Chen Q.-H., Yu J.-Q., Zheng R.-Q. (2017). Early initiation of low-dose hydrocortisone treatment for septic shock in adults: A randomized clinical trial. Am. J. Emerg. Med..

[27] Annane D., Renault A., Brun-Buisson C., Megarbane B., Quenot J.-P., Siami S., Cariou A., Forceville X., Schwebel C., Martin C. (2018). Hydrocortisone plus Fludrocortisone for Adults with Septic Shock. N. Engl. J. Med..

[28] Annane D., Sébille V., Charpentier C., Bollaert P.-E., François B., Korach J.-M., Capellier G., Cohen Y., Azoulay E., Troché G. (2002). Effect of Treatment with Low Doses of Hydrocortisone and Fludrocortisone on Mortality in Patients with Septic Shock. JAMA.

[29] Cicarelli D.D., Vieira J.E., Benseñor F.E.M. (2007). Early dexamethasone treatment for septic shock patients: A prospective randomized clinical trial. Sao Paulo Med. J..

[30] Schumer W. (1976). Steroids in the treatment of clinical septic shock. Ann. Surg..

[31] Bone R.C., Fisher C.J., Clemmer T.P., Slotman G.J., Metz C.A., Balk R.A., The Methylprednisolone Severe Sepsis Study Group (1987). A Controlled Clinical Trial of High-Dose Methylprednisolone in the Treatment of Severe Sepsis and Septic Shock. N. Engl. J. Med..

[32] Luce J.M., Montgomery A.B., Marks J.D., Turner J., Metz C.A., Murray J.F. (1988). Ineffectiveness of High-dose Methylprednisolone in Preventing Parenchymal Lung Injury and Improving Mortality in Patients with Septic Shock. Am. Rev. Respir. Dis..

[33] Veterans Administration Systemic Sepsis Cooperative Study Group (1987). Effect of high-dose glucocorticoid therapy on mortality in patients with clinical signs of systemic sepsis. N. Engl. J. Med..

[34] Yildiz O., Doganay M., Aygen B., Güven M., Klelestimur F., Tutuş A. (2002). Physiological-dose steroid therapy in sepsis [ISRCTN36253388]. Crit. Care.

[35] Yildiz O., Tanriverdi F., Simsek S., Aygen B., Kelestimur F. (2011). The effects of moderate-dose steroid therapy in sepsis: A placebo-controlled, randomized study. J. Res. Med. Sci..

[36] Minneci P., Deans K., Eichacker P., Natanson C. (2009). The effects of steroids during sepsis depend on dose and severity of illness: An updated meta-analysis. Clin. Microbiol. Infect..

[37] Ahmed M.H., Hassan A. (2020). Dexamethasone for the Treatment of Coronavirus Disease (COVID-19): A Review. SN Compr. Clin. Med..

[38] Horby P., Lim W.S., Emberson J.R., Mafham M., Bell J.L., Linsell L., Staplin N., Brightling C., Ustianowski A., Elmahi E. (2021). Dexamethasone in hospitalized patients with Covid-19. N. Engl. J. Med..

