# Supplementary material for: New Evidence of Potential Benefits of Dexamethasone and Added on Therapy of Fludrocortisone on Clinical Outcomes of Corticosteroid in Sepsis Patients: A Systematic Review and Meta-Analysis
Source: J Pers Med. 2021 Jun 11;11(6):544. doi: 10.3390/jpm11060544 (PMC8231131; doi:10.3390/jpm11060544)
Supplement: Supplementary file 1 [file jpm-11-00544-s001.zip › jpm-1237891-supplementary.pdf]

Supplement 1. Quality assessment of included studies of randomized controlled trials

	Random sequence generation	Allocation concealment	Blinding of participants and personnel	Blinding outcome assessment	Incomplete outcome data	Selective reporting	Other bias
Annane D et al (2002)	L	L	L	L	L	L	L
Annane D et al (2018)	L	L	L	L	L	L	U
Bollaert PE et al (1998)	L	L	L	L	L	L	U
Bone RC et al (1987)	L	L	H	L	L	U	L
Briegel J et al (1999)	U	U	L	L	L	U	L
Briegel J et al (2001)	U	U	H	L	L	L	H
Cicarelli DD et al (2007)	L	U	L	L	U	L	L
Confalonieri M et al (2005)	L	L	L	L	L	L	L
Keh D et al (2016)	L	L	L	L	L	L	L
Kaufmann I et al (2008)	U	U	L	L	L	L	L
Luce JM et al (1988)	L	L	L	L	L	L	L
Lv QQ et al (2017)	L	H	L	L	L	L	L
Moreno R et al (2011)	L	L	L	L	L	L	L
Oppert M et al (2005)	U	L	L	L	U	U	L

Schelling G et al (2001)	U	U	L	L	L	L	L
Schumer W et al (1976)	H	H	L	L	L	H	H
Sprung CL et al (2008)	L	L	L	L	U	L	U
Tongyoo et al (2016)	L	L	L	L	L	U	L
VASSCSG (1987)	L	L	L	L	U	U	U
Venkatesh B et al (2018)	L	L	L	L	L	L	L
Yildiz O et al (2002)	L	L	L	L	L	U	U
Yildiz O et al (2011)	L	L	L	L	L	U	U

a)

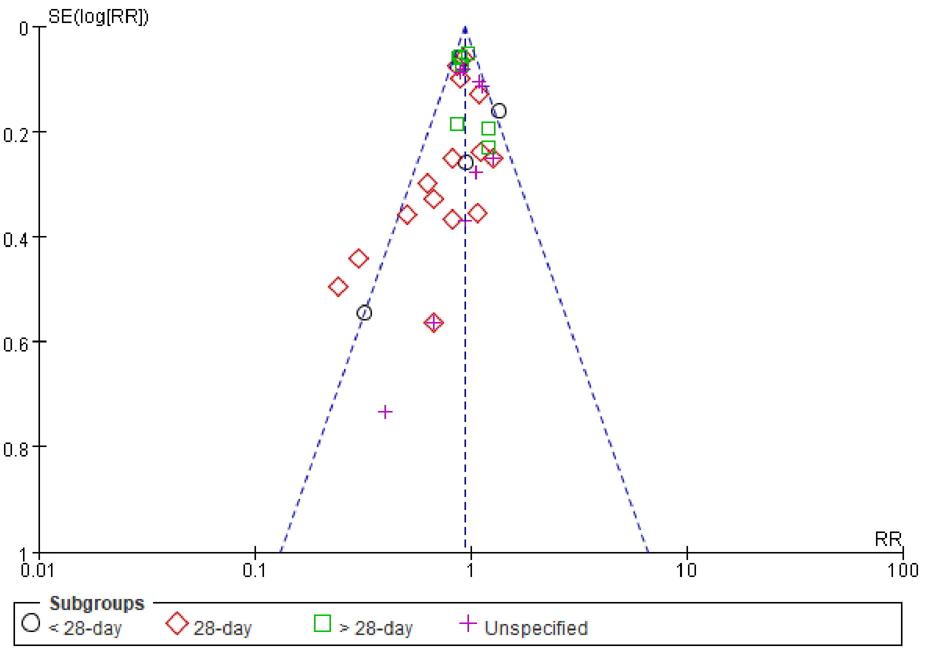

b)

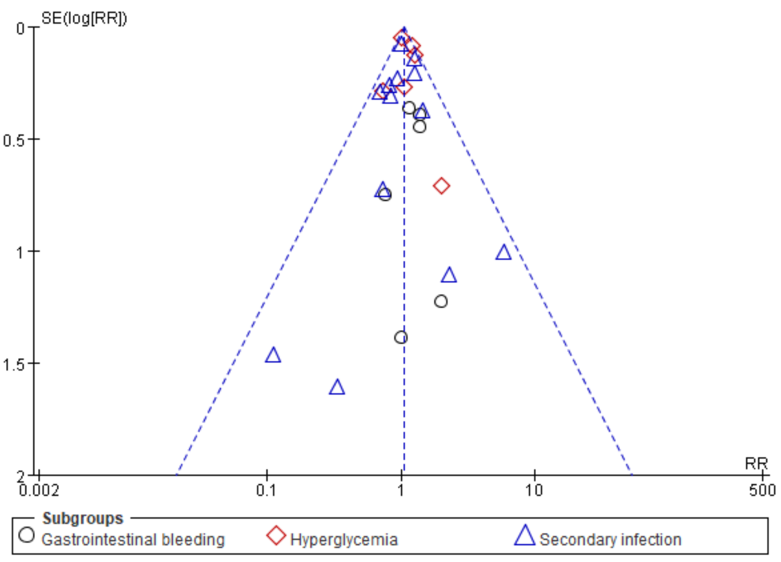

Supplementary Figure S1. Funnel plots of the study outcomes; a) mortality, and b) adverse events.
